# Endothelial-derived angiocrine factors as instructors of embryonic development

**DOI:** 10.3389/fcell.2023.1172114

**Published:** 2023-06-29

**Authors:** Daniel Bishop, Quenten Schwarz, Sophie Wiszniak

**Affiliations:** Centre for Cancer Biology, University of South Australia, Adelaide, SA, Australia

**Keywords:** angiocrine, endothelial, development, embryonic development, blood vessel, signaling

## Abstract

Blood vessels are well-known to play roles in organ development and repair, primarily owing to their fundamental function in delivering oxygen and nutrients to tissues to promote their growth and homeostasis. Endothelial cells however are not merely passive conduits for carrying blood. There is now evidence that endothelial cells of the vasculature actively regulate tissue-specific development, morphogenesis and organ function, as well as playing roles in disease and cancer. Angiocrine factors are growth factors, cytokines, signaling molecules or other regulators produced directly from endothelial cells to instruct a diverse range of signaling outcomes in the cellular microenvironment, and are critical mediators of the vascular control of organ function. The roles of angiocrine signaling are only beginning to be uncovered in diverse fields such as homeostasis, regeneration, organogenesis, stem-cell maintenance, cell differentiation and tumour growth. While in some cases the specific angiocrine factor involved in these processes has been identified, in many cases the molecular identity of the angiocrine factor(s) remain to be discovered, even though the importance of angiocrine signaling has been implicated. In this review, we will specifically focus on roles for endothelial-derived angiocrine signaling in instructing tissue morphogenesis and organogenesis during embryonic and perinatal development.

## Introduction

**FIGURE 1:**
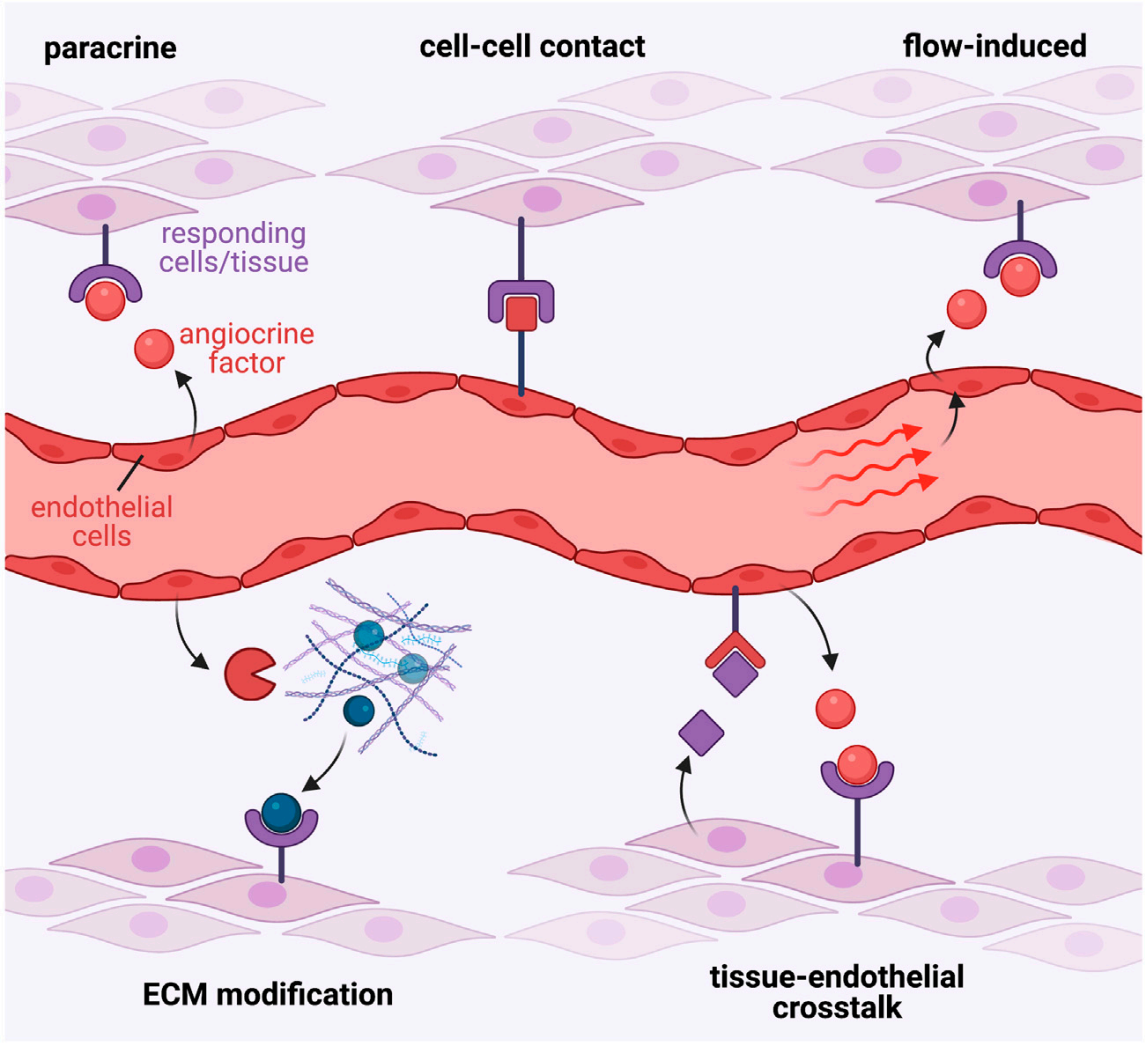
Diverse modes of angiocrine signalling. Endothelial-derived angiocrine factors signal to target tissues and cell-types via diverse molecular mechanisms. These may include, but are not limited to: 1. Simple paracrine signalling involving endothelial secretion of a soluble factor that directly acts on responding cells; 2. Direct cell-cell contact mediated mechanisms that facilitate signalling between endothelial-derived ligands and their corresponding receptors on target cells; 3. Blood flow and shear force-mediated stimulation of the endothelium to induce expression of angiocrine signals; 4. Release of angiocrine factors that modify the surrounding extra-cellular matrix to elicit direct effects, or in-direct signalling via subsequent release of ECM-bound molecules; or 5. Complex tissue-endothelial cell cross-talk interactions, where the target tissue may release signalling molecules to induce angiocrine factor release from endothelial cells, which then in turn signal back to the target tissue to induce the desired response.

The cardiovascular network forms during early embryogenesis, and is essential for the delivery of oxygen and nutrients, as well as removal of waste products from cells and tissues, which is essential for correct growth of the developing embryo. The vasculature forms primarily through angiogenesis, whereby endothelial cells, which make up the inner lining of vessels, respond to angiogenic signals which stimulate their proliferation, sprouting and remodeling into an organized network of arteries, veins and capillaries (Potente et al., 2011). In addition to their requisite role in transportation of blood around the organism, there is now evidence that blood vessels actively contribute to and direct morphogenesis of the embryo. Endothelial cells can actively produce signaling molecules which act on surrounding cells and tissues in a paracrine or juxtacrine manner, which has now emerged as a fundamental mechanism of tissue morphogenesis. These endothelial-derived factors are known as “angiocrine” factors. The term angiocrine was first coined in 2010 (Butler et al., 2010) to highlight the biological significance of instructive factors produced by endothelial cells, but endothelial signaling has been appreciated as an important mechanism instructing tissue morphogenesis for some time before this (Cleaver and Melton, 2003). Our understanding of the diverse modes of angiocrine signaling are continually evolving, from simple paracrine secretion, to cell-cell contact dependent roles, as well as complex tissue-endothelial feedback loops (Figure 1). Furthermore, as well as vascular endothelium, cardiac endothelium (endocardium) and lymphatic endothelium have also been shown to produce factors which influence the development of surrounding cells and tissues. In this review we explore roles for angiocrine signaling encompassing all these endothelial cell types.

Over recent years, with the advent of new technologies such as single-cell analysis, it has now become possible to interrogate the molecular interactions between endothelial cells and their microenvironment, and in turn how endothelial cells influence organogenesis in a tissue-specific manner. While in some cases tissue-specific vasculature has long been known to direct development and differentiation of surrounding cells, the molecular identity of the specific endothelial-derived angiocrine factors directing these processes have often remained elusive. Given the recent emergence of new technologies in transcriptomics, proteomics, and genetic engineering, many of these previously uncharacterised angiocrine factors are now beginning to be identified and validated *in vivo* in animal models.

Other recent reviews (Pasquier et al., 2020; Gomez-Salinero et al., 2021; Chen and Ding, 2022) have documented roles for angiocrine factors in tissue regeneration following injury, adult stem cell niches and disease such as cancer. In this review, we will focus on the tissue-specific roles of angiocrine factors in directing the development, differentiation and morphogenesis of diverse organ and tissue systems during embryogenesis and the early perinatal period. We will discuss organ systems in which angiocrine signaling has been demonstrated to play a role; both where the angiocrine factors involved have been molecularly identified, as well as those for which the specific factors remain to be discovered. A summary of the known and newly identified angiocrine factors with *in vivo* evidence for roles in instructing normal embryo development are presented in Table 1.

**TABLE 1:** Angiocrine factors involved in embryonic development. Summary of angiocrine factors involved in development, their target organ/tissues, and a brief description of the functional role of angiocrine signalling. This table encompasses a selection of angiocrine factors that have demonstrated *in vivo* evidence of function, and is not an exhaustive list of all angiocrine factors involved in development.

Target organ/tissue	Angiocrine factor	Function	References
Vessel wall	PDGF-B	Recruits peri-vascular cells in blood vessel wall assembly	Zerwes and Risau (1987), Hirschi et al. (1998), Enge et al. (2002), Bjarnegard et al. (2004)
Vessel wall	Jag1	Promotes vascular smooth muscle differentiation	High et al. (2007), High et al. (2008)
Lymphatics	Vegfc	Mediates lymphatic patterning	Chiang et al. (2023)
Heart	Nrg1	Cardiac trabeculation	Gassmann et al. (1995), Lee et al. (1995), Meyer and Birchmeier (1995), Kramer et al. (1996), Grego-Bessa et al. (2007), Liu et al. (2010), Samsa et al. (2015), Del Monte-Nieto et al. (2018)
Heart	Wnt4	Induces BMP2 expression in myocardium during heart valve formation	Wang et al. (2013)
Heart	HB-EGF	Potentiates hyperproliferation of valve mesenchyme during heart valve formation	Iwamoto et al. (2003), Jackson et al. (2003), MacGrogan et al. (2016)
Heart	Wnt9b	Promotes mesenchymal cell condensation in heart valve formation	Goddard et al. (2017)
Heart	RELN	Lymphangiocrine factor; promotes cardiomyocyte proliferation and heart growth	Liu et al. (2020)
Skeletal	Mmp9	Regulates cartilage resorption in endochondral ossification	Romeo et al. (2019)
Skeletal	Noggin	BMP antagonist, essential for correct bone and growth plate formation	Ramasamy et al. (2014)
Skeletal	BMP4	Promotes intramembranous ossification in the opercles, induces Runx2 expression	Uemura et al. (2016)
Skeletal	IGF1	Drives proliferation of Meckel’s cartilage chondrocytes in mandible development	Marchant et al. (2020)
Liver	Wnt ligands	Regulates the polarization of hepatocytes, vascular/biliary network patterning	Leibing et al. (2018), Zhu et al. (2022)
Pancreas	EGFL7	Regulates proliferation/maintenance/expansion of pancreatic progenitors	Kao et al. (2015)
Lung	Sash1/NO	Activates eNOS, induces alveolar epithelial cell maturation	Coulombe et al. (2019)
Lung	HGF	Lung epithelial cell differentiation	Yamamoto et al. (2007), Yao et al. (2017)
Kidney	MMP2	Stretch dependent expression, regulates glomerular formation	Serluca et al. (2002)
Nervous System	BMP4, BMP7	Regulates differentiation of sympathetic neurons	Reissmann et al. (1996), Saito et al. (2012)
Nervous System	TGFβ1	Regulates differentiation of oligodendrocytes in spinal cord	Paredes et al. (2021)
Haematopoiesis	Wnt2	Haematopoietic colonization and HSC proliferation	Liu et al. (2022)

### Cardiovascular system

One of the first identified angiocrine factors was platelet-derived growth factor B (PDGF-B), known for its role in mediating pericytes recruitment and blood vessel wall assembly. Early work showed PDGF-B is secreted from the basal surface of endothelial cells and is chemotactic for smooth muscle cells (Zerwes and Risau, 1987; Hirschi et al., 1998), which express its receptor PDGFRβ (Shinbrot et al., 1994). PDGF-B and PDGFRβ knockout mouse embryos exhibit vessel dilation, haemorrhaging and capillary microaneurysms attributed to a lack of microvascular pericytes coverage (Leveen et al., 1994; Lindahl et al., 1997; Hellstrom et al., 1999), with endothelial-specific PDGF-B conditional knockout also causing impaired pericytes recruitment to blood vessels (Enge et al., 2002; Bjarnegard et al., 2004), suggesting angiocrine PDGF-B is required for pericytes recruitment *in vivo*. Angiopoietin signaling has also been implicated in vessel wall assembly. The tyrosine kinase receptor Tie2 is expressed on endothelial cells, and is stimulated by its ligand angiopoietin-1, which is secreted by the mesenchyme surrounding vessels (Davis et al., 1996). Angiopoietin-2 is an additional ligand for Tie2, which was initially described as an antagonist (Maisonpierre et al., 1997), however it does exhibit agonist properties in specific cellular contexts (Akwii et al., 2019). Mouse embryos lacking angiopoietin-1 exhibit vascular defects attributed to loss of association between endothelial cells and pericytes (Suri et al., 1996), suggesting Tie2 activation by angiopoietin-1 may trigger release of a chemo attractant by endothelial cells to recruit perivascular cells. Later studies demonstrated that angiopoietin-1 stimulates endothelial cells to upregulate expression of heparin binding EGF-like growth factor (HB-EGF) (Iivanainen et al., 2003) and hepatocyte growth factor (HGF) (Kobayashi et al., 2006), both of which stimulate smooth muscle cell migration towards endothelial cells, and hence represent additional angiocrine factors important for pericytes recruitment.

A recent study using an *in vitro* 3D pericytes/endothelial co-culturing system has also revealed the complexity of endothelial-derived factors in pericytes recruitment and capillary network formation. Factors such as TGF-β1 were shown to prime pericytes to respond to PDGFs and ET-1 which drive pericytes invasion, with HB-EGF rather stimulating pericytes proliferation (Kemp et al., 2020). However the interactions between these factors in pericytes function *in vivo* remain to be fully explored.

The influence of endothelial-derived factors on vascular smooth muscle differentiation is particularly important in formation of large calibre vessels, such as the aorta and pharyngeal arch arteries, which are surrounded by multiple concentric layers of smooth muscle cells (Bergwerff et al., 1996). Notch signaling is known to play cell-autonomous roles in endothelial cells to control multiple aspects of vascular development (Akil et al., 2021), however this pathway is also utilized in an angiocrine fashion to signal to surrounding cell-types, such as smooth muscle cells, to influence their development and differentiation. Endothelial-specific knockout of the Notch ligand Jagged 1 (Jag1) in mouse embryos leads to loss of vascular smooth muscle coating on vessels, resulting in cardiovascular defects including dilated vessels and haemorrhaging (High et al., 2008). Furthermore, interruption of downstream Notch signaling in neural crest cells, which are the progenitors of vascular smooth muscle around the pharyngeal arch arteries, inhibits smooth muscle differentiation, leading to arch artery remodeling defects (High et al., 2007). Together, this demonstrates angiocrine Jag1 is required to signal to Notch receptors on neighbouring smooth muscle progenitors to promote vascular smooth muscle differentiation in a direct cell-cell contact dependent manner. A recent study also demonstrated that in the pharyngeal arch arteries endothelial integrin α5β1, the receptor for fibronectin, is required for appropriate differentiation of neural crest-derived vascular smooth muscle cells, likely via a Notch independent mechanism (Warkala et al., 2021). Blood vessel-derived factors are also involved in correctly patterning other vascular beds such as the dermal lymphatic vasculature, with the blood endothelial-specific transcription factor Sox7 shown to repress the expression of the major lymphangiogenic growth factor VEGFC (Chiang et al., 2023). Endothelial-specific knockout of Sox7 leads to defective lymphatic patterning and oedema, consistent with the phenotype of VEGFC overexpression (Chiang et al., 2023), suggesting Sox7 is an important regulator of angiocrine signals, such as VEGFC, that pattern the dermal lymphatics.

Endocardial-derived angiocrine signals are also important for aspects of heart development, such as ventricular trabeculation and valve formation (Kim et al., 2021). Trabeculae are myocardial protrusions that project into the lumen of the cardiac ventricles, which function to increase cardiac output, as well as the surface area of the embryonic myocardium for nutrient and oxygen uptake (Sedmera et al., 2000). Trabeculae are lined by an outer layer of endocardial endothelium, and this close apposition of endocardium and myocardium facilitates intercellular signaling events that are essential for their correct formation, patterning and growth (Qu et al., 2022). Neuregulin 1 (Nrg1) is one such signaling molecule that is expressed by and secreted from the endocardium to signal to ErbB2/ErbB4 receptor complexes expressed on myocardial cells to initiate trabeculation (Odiete et al., 2012). Early work demonstrated that knockout of Nrg1, ErbB2, or ErbB4 in mouse embryos leads to a loss of trabeculae (Gassmann et al., 1995; Lee et al., 1995; Meyer and Birchmeier, 1995; Kramer et al., 1996). The paracrine signaling of Nrg1 to ErbB receptor complexes stimulates proliferation of trabecular cardiomyocytes, as well as inducing their directed migration to initiate trabeculae formation (Liu et al., 2010). Acting upstream, Notch1 activation in the endocardium drives transcription of its direct target EphrinB2, which in turn upregulates Nrg1 expression (Grego-Bessa et al., 2007; Samsa et al., 2015). However, recent studies suggest a reciprocal interaction between Notch1 and Nrg1 in cardiac trabeculation, whereby Notch1 signaling promotes ECM degradation, and Nrg1 invokes ECM synthesis of the cardiac jelly, which is located between the endocardial and cardiomyocyte layers of the forming trabeculae (Del Monte-Nieto et al., 2018), suggesting a precise balance of ECM regulation by endocardial-derived factors is essential for proper trabeculation. Defective trabeculation, as well as malformation of the coronary arteries, can lead to phenotypes such as ventricular non-compaction (Weiford et al., 2004). A recent study has used single-cell RNA sequencing to identify several angiocrine factors, such as Col15a1, secreted from endocardium and/or coronary vessels that may potentially impact ventricular compaction in normal development, as well as non-compaction pathologies (Rhee et al., 2021).

**FIGURE 2:**
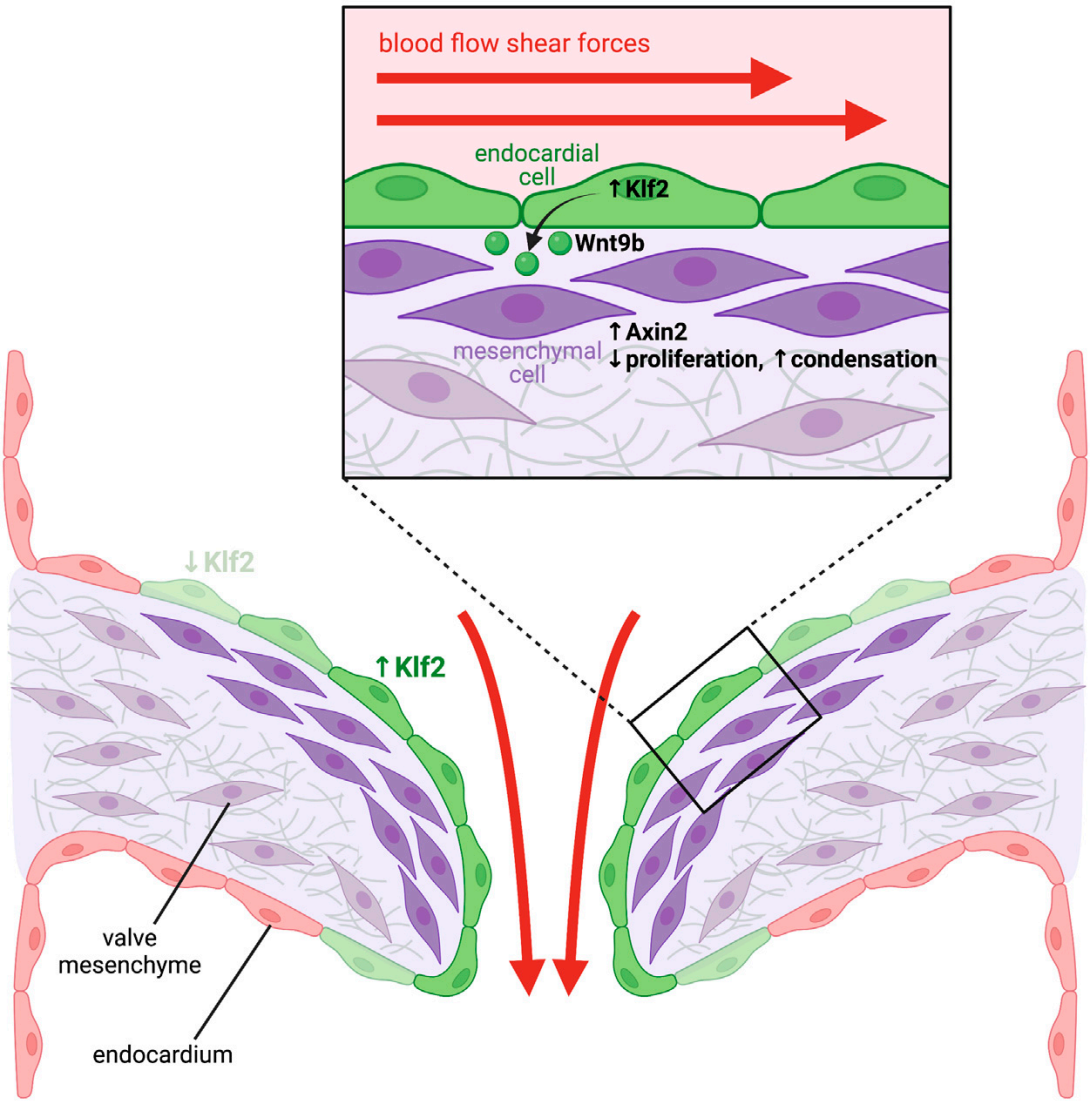
Blood-flow mediated haemodynamic shear forces stimulate endocardial cells to release angiocrine factors that instruct heart valve development. High shear forces stimulate expression of the shear force-responsive transcription factor Klf2 in endocardial cells located in the area of the forming heart valves (green cells). Klf2 then induces expression of the secreted ligand Wnt9b, which acts in a paracrine manner on mesenchymal cells located within the underlying cardiac cushions. Activation of the Wnt signaling pathway in these cells stimulates expression of Wnt target genes, such as Axin2 (dark purple cells), which limits proliferation and induces mesenchymal cell condensation, which is essential for remodeling of the cardiac cushions into mature valve leaflets.

Endocardium also plays an important role in heart valve formation, including both the semilunar outflow tract valves, as well as the atrio-ventricular (AV) valves. Valve formation is preceded by endothelial-to-mesenchymal transition (EndoMT) of a subpopulation of endocardial cells to form valve interstitial cells which expand the cardiac jelly located between the endocardium and myocardium (O'Donnell and Yutzey, 2020). These endocardial cushions subsequently remodel and elongate to form the mature valve leaflets (O'Donnell and Yutzey, 2020). An interplay between Notch signalling in the endocardium and BMP signaling in the myocardium has been implicated in regulating both initial EndoMT and valve maturation. Notch1 is expressed in the endocardium where it transcriptionally regulates Snail (Timmerman et al., 2004), a transcription factor that represses VE-cadherin expression, hence reducing cell-cell adhesions between endocardial cells and promoting EndoMT (Alvandi and Bischoff, 2021). Global loss of Notch signalling via Notch1 or RBPJκ knockout causes impaired EndoMT in the AV valve primordium (Timmerman et al., 2004), as does endothelial-specific knockout of Notch1 (Hofmann et al., 2012), whereas constitutively active Notch expression in the endocardium results in ectopic EndoMT (Luna-Zurita et al., 2010). Endocardial Notch ligands Jag1 and Dll4 have both been implicated in regulating Notch1 activity, however endothelial-specific knockout of Jag1 has produced differing phenotypes; in some cases inhibiting EndoMT and producing hypocellular endocardial cushions (Hofmann et al., 2012; Wang et al., 2013), while other studies have shown it to be dispensable for EndoMT (MacGrogan et al., 2016). Myocardial-derived BMP2 has also been implicated in inducing EndoMT of the adjacent endocardium. BMP2 is expressed in a discreet domain of myocardium overlying the AV primordium, and myocardial-specific knockout of BMP2, or endocardial-specific knockout of the BMP receptor Bmpr1a result in failed cushion formation, indicating BMP2 signals directly to the endocardium to induce EndoMT (Ma et al., 2005). Another study suggests BMP2 signaling to the endocardium may directly regulate Notch signaling activity by transcriptionally activating Jag1 (Papoutsi et al., 2018). The integration of Notch and BMP signaling, as well as the cell-cell interactions between endocardium and myocardium that orchestrate valve development are complex, but importantly several studies have implicated endocardial-derived angiocrine factors in this process. One study demonstrated that endocardial knockout of Notch1 or Jag1 using a Nfatc1-Cre driver led to a loss of Wnt4 expression in the endocardium, and a reduction of BMP2 expression in the myocardium (Wang et al., 2013). Similarly, inhibition of Wnt signaling using a small molecule inhibitor reduced BMP2 expression in the myocardium, and treatment of *Nfatc1-Cre; Notch1* ^*flox*^ embryos with recombinant Wnt4 was able to increase BMP2 expression in the myocardium (Wang et al., 2013). This suggests that Notch signaling in the endocardium induces expression of Wnt4 which then acts in an angiocrine manner to upregulate BMP2 in the myocardium. Another study demonstrated that endocardial knockout of Jag1 did not inhibit the initial processes of EndoMT, and rather implicated Dll4 as the endocardial Notch ligand important for initiating EndoMT (MacGrogan et al., 2016). In this study, endocardial Jag1 knockout caused enlarged valve leaflets and hyperproliferation of the valve mesenchyme due to excessive BMP-driven SMAD signaling, suggesting Jag1-Notch1 signaling is required to restrict mesenchyme proliferation post-EndoMT. HB-EGF was identified as a direct Notch transcriptional target in the endocardium, and EGFR activation by HB-EGF was shown to inhibit SMAD signaling, thus potentiating hyperproliferation of the valve mesenchyme (MacGrogan et al., 2016). In agreeance with this, knockout of HB-EGF, which is almost exclusively expressed by the valve endocardium, leads to enlarged hyperproliferative valves and dramatic increases in activated SMAD (Iwamoto et al., 2003; Jackson et al., 2003). Recently, mass-spectrometry has been used to characterise the secretome of mouse embryonic endocardial cells upon Notch stimulation and inhibition (Torregrosa-Carrion et al., 2019), and thus represents a valuable resource for the identification of novel candidate angiocrine factors involved in valve and cardiac development that await *in vivo* characterisation. Hemodynamic shear forces have also been implicated in stimulating the endocardium to secrete angiocrine factors regulating valve morphogenesis (Figure 2). Endocardial-specific knockout of KLF2, a shear-responsive transcription factor, causes enlarged endocardial cushions and valves with excessive mesenchymal proliferation (Goddard et al., 2017). Wnt9b was identified as a KLF2-responsive gene, which is expressed exclusively by the endocardium and acts in a paracrine manner on adjacent valve mesenchymal cells to activate canonical Wnt signalling and promote correct mesenchymal cell condensation (Goddard et al., 2017). Endocardial-specific knockout of Wnt9b exhibits identical valve defects to KLF2 knockout, suggesting Wnt9b is a critical angiocrine factor mediating this process (Goddard et al., 2017). Furthermore, using zebrafish models with reduced or absent blood flow, the authors demonstrate hemodynamic shear forces are essential to upregulate expression of KLF2 and Wnt9b in the valve endocardium (Goddard et al., 2017).

Lymphatic endothelial cells have also recently been shown to secrete lymphangiocrine signals to regulate heart development. Cardiac lymphatics appear during the later stages of heart development, where they expand over the dorsal surface of the heart and into the myocardium (Klotz et al., 2015). Ablation of cardiac lymphatics reduces heart size and cardiomyocyte proliferation *in vivo*, with lymphatic endothelial cell conditioned medium shown to promote cardiomyocyte proliferation *in vitro* (Liu et al., 2020), suggesting a secreted factor is responsible. Mass spectrometry analysis of conditioned media identified RELN, an ECM protein that binds to and signals through integrin-β1 receptors, as a candidate factor (Liu et al., 2020). Lymphatic endothelial specific knockout of RELN leads to reduced heart growth and cardiomyocyte proliferation *in vivo*, identifying RELN as a novel lymphangiocrine factor important for correct heart growth (Liu et al., 2020).

### Skeletal system

Endochondral bone development, that forms the bulk of the long bones, relies on the initial formation of a hyaline cartilage template, which is subsequently replaced by bone [reviewed in (Long and Ornitz, 2013)]. Briefly, during ossification resting chondrocytes in the avascular cartilage growth plate proliferate then become hypertrophic, which triggers the growth of blood vessels into the cartilage, while concomitantly inducing chondrocyte apoptosis. Blood vessel angiogenesis is accompanied by the invasion of osteoblasts which replace the cartilage template with mineralised bone. Hence, the precise coupling of cartilage growth/regression (chondrogenesis) and osteogenesis with angiogenesis is critically important for the formation of ossified bone. Early work hypothesised that blood vessels may play instructive roles in regulating chondrocyte hypertrophy and late-stage chondrocyte differentiation. For example, *in vitro* co-culture experiments demonstrated that proteases secreted from endothelial cells induced rapid hypertrophy and late-differentiation of chondrocytes (Babarina et al., 2001). *In vivo* studies have also demonstrated that blocking VEGF-dependent blood vessel invasion into bone inhibits hypertrophic chondrocytes from undergoing cell death, resulting in enlarged growth plates, further suggesting endothelial cells may regulate chondrocyte late-differentiation (Gerber et al., 1999). A recent study definitively identified MMP9 as an endothelial-derived protease regulating cartilage resorption in the growth plate (Romeo et al., 2019). Endothelial-specific deletion of Mmp9 in early postnatal mice lead to enlarged growth plates and increased expression of hypertrophic chondrocyte markers such as collagen-X and MMP13 (Romeo et al., 2019). Surprisingly, deletion of MMP9 in the osteoclast lineage, or depletion of osteoclasts by genetic or pharmacological means, did not alter growth plate size, suggesting osteoclasts are dispensable for chondrocyte resorption (Romeo et al., 2019), and thus further highlighting the previously unrecognised role for blood vessels in removal of cartilage matrix. Blood vessels also play important roles in osteoblast development. Bone endothelial cells can be classified into specialised subtypes based on distinct localisation, morphology and differential gene expression, namely, type H (high expression of blood vessel markers CD31 and endomucin) and type L populations (low expression of CD31 and endomucin), with osteoprogenitor selectively positioned around type H endothelium (Kusumbe et al., 2014), suggesting these vessels may provide niche signals for perivascular osteoprogenitor. Furthermore, Notch signaling in type H endothelium mediates the angiocrine secretion of Noggin, a BMP antagonist, which is essential for correct bone and growth plate formation (Ramasamy et al., 2014), however which cell type(s) angiocrine Noggin specifically acts on, i.e., osteoprogenitor or growth plate chondrocytes, remain to be elucidated. Lymphatic vessels have also been identified in long bones, with elegant tissue-specific genetic mouse models demonstrating that lymphangiocrine CXCL12 promotes expansion of Myh11-positive pericytes which differentiate into perivascular osteoblasts to drive bone regeneration following genotoxic stress (Biswas et al., 2023). Myh11-positive pericytes are located close to vessels in early postnatal bone development (Biswas et al., 2023), however the role of lymphangiocrine factors in normal bone development during embryogenesis remains to be investigated. *In vitro* studies have also demonstrated a direct interaction between human umbilical vein endothelial cells (HUVEC) and human bone marrow osteoprogenitor stromal cells (HBMSC) mediated by gap junctions involving connexin-43, with this interaction essential to drive osteoprogenitor differentiation, and upregulation of osteoblast markers (Villars et al., 2002).

In contrast to the long bones of the skeleton, flat bones such as in the face and skull as well as the clavicles are formed through intramembranous ossification, whereby mesenchymal progenitors differentiate directly into bone without the requirement for an intermediary cartilage template (Ko and Sumner, 2021). Blood vessels are intimately associated with forming intramembranous bones (Thompson et al., 1989), hence the angiocrine influence of endothelium on intramembranous ossification has been investigated in several studies. For example, vessel-derived endothelin-1 has been shown to induce proliferation and osteoblastic differentiation of *ex vivo* cultured rat fetal calvarial cells (von Schroeder et al., 2003). Blood vessels have also been shown to be a major source of the bone-promoting morphogen BMP-2 during distraction osteogenesis, which is a bone healing process considered analogous to developmental intramembranous ossification (Matsubara et al., 2012). Furthermore, a study in zebrafish embryos demonstrated that BMP-4 secreted from endothelial cells, under the transcriptional influence of Yap/Taz, is required for inducing Runx2 expression in pre-osteoblasts and promoting correct intramembranous ossification of the opercula bones (Uemura et al., 2016).

**FIGURE 3:**
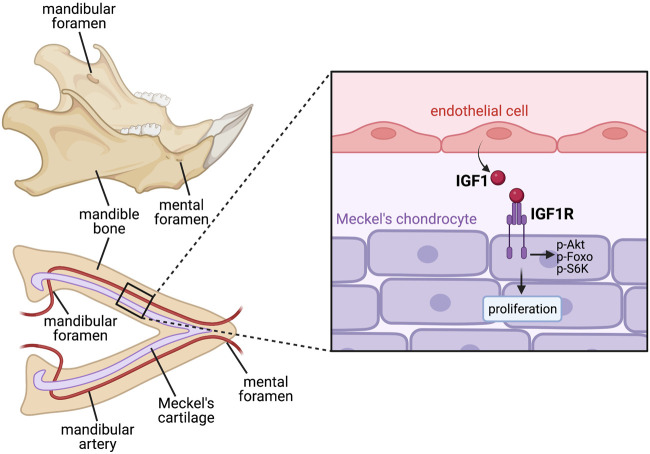
Paracrine angiocrine signalling promotes Meckel’s cartilage proliferation to drive jaw growth during development. Jaw bone formation is preceded by the initial formation of the arrowhead-shaped Meckel’s cartilage, which acts as a scaffold for the mandible bone to later ossify around. This size and shape of Meckel’s cartilage therefore dictates the shape of the jaw bone later in development. Meckel’s cartilage lies in close apposition with the mandibular artery, which releases angiocrine IGF1 to act in a paracrine manner on IGF1R on Meckel’s chondrocytes, inducing their proliferation and promoting appropriate jaw lengthening prior to bone ossification. Since ossification of the mandible bone occurs around the already formed mandibular artery, foramen (holes) are present in mature jaw bone which allow passage of the artery and continued blood flow through the bone.

Angiocrine factors have also been shown to influence cartilage development. Mouse models with disrupted craniofacial vasculature, particularly malformation of the mandibular artery, exhibit defective growth and proliferation of Meckel’s cartilage in the lower jaw, with blood vessels shown to secrete soluble factors that can promote Meckel’s chondrocyte proliferation, suggesting an angiocrine requirement for blood vessels in embryonic jaw development (Wiszniak et al., 2015). Further work identified IGF-1 as a definitive angiocrine factor secreted from blood vessels, with endothelial-specific removal of IGF-1 disrupting Meckel’s cartilage proliferation and inhibiting correct jaw extension (Marchant et al., 2020) (Figure 3).

Endothelial cells also play important roles in tooth mineralisation. Periodontal tip-like endothelial cells located within the dental pulp supply phosphate to odontoblasts, which is essential for their ability to mineralise dentin (Matsubara et al., 2022). Additionally, TGFβ and PTN were identified as endothelial-expressed factors that were able to promote expression of dentinogenesis markers in the dental pulp, hence suggesting tooth mineralisation may be mediated by angiocrine mechanisms (Matsubara et al., 2022).

### Liver

The liver is a vitally important metabolic organ that is responsible for many essential processes. Structurally, the lobes of the liver are divided into thousands of functional units known as lobules that consist primarily of hepatocytes. These lobules are surrounded by a rich vascular network that is derived from both the hepatic artery and portal vein (Saxena et al., 1999). The smaller vessels between the lobules are known as sinusoids which drain blood toward the central vein in the middle of each lobule (Lautt, 1977). This dense network of vessels has been shown to host a vascular niche that secretes angiocrine factors necessary for both embryogenesis and regeneration (Ding et al., 2010).

Initial discoveries of the potential role for angiocrine signaling in this organ system transpired by impairing VEGF signaling and observing the subsequent impact on liver development. In mice lacking the VEGF receptor Flk1 (Shalaby et al., 1995), the proliferation and expansion of hepatic cells into the septum transversum mesenchyme was impaired, however the initial budding of the hepatic endoderm proceeded normally (Matsumoto et al., 2001). When *Flk1* ^*−/−*^ derived liver explants were cultured *in vitro,* the growth of the hepatic domain was also significantly reduced when compared to wildtype explants (Matsumoto et al., 2001). Additionally, inhibition of endothelial growth in wildtype liver explants with the anti-angiogenic recombinant NK4 protein [consisting of four kringle domains of HGF, and exhibiting structural similarity to angiostatin (Date et al., 1998)] similarly reduced hepatic growth, via a HGF signaling-independent mechanism (Matsumoto et al., 2001). Interestingly, primary adult hepatocyte growth in culture is enhanced by co-culture with sinusoidal endothelial cells that are stimulated with VEGF (LeCouter et al., 2003). VEGFR-1 activation was shown to stimulate endothelial expression of HGF, which in turn induced hepatocyte growth, implicating HGF as a sinusoidal derived angiocrine factor in the adult liver (LeCouter et al., 2003). A recent study provided conclusive evidence of an angiocrine role for HGF in liver growth, with liver sinusoidal endothelial cell-specific knockout of HGF causing decreased hepatocyte proliferation and regeneration post-hepatectomy, although fetal liver development was unaffected (Zhang X. J. et al., 2020).

**FIGURE 4:**
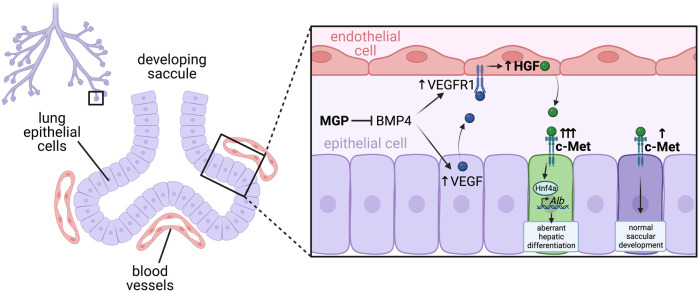
Precise balance of angiocrine HGF regulates saccular development in the lung. Blood vessels sit in close apposition to lung epithelial cells in the developing saccules. Matrix Gla Protein (MGP) is an antagonist of BMP, and acts to regulate activity of BMP4 in the developing lung. Knockout of MGP leads to excessive BMP signaling, which upregulates VEGF expression from lung epithelial cells and VEGFR1 expression on endothelial cells, which together results in increased VEGFR signaling in endothelial cells. This stimulates angiocrine expression of hepatocyte growth factor (HGF) which then signals to c-Met receptors on lung epithelial cells. In the case of excessive HGF and c-Met signaling, this induces expression of the transcription factor Hnf4a, which subsequently binds to and stimulates transcription of its direct target Albumin, causing aberrant hepatic differentiation of lung epithelial cells. However, in cases of adequately regulated c-Met signaling, HGF acts to promote normal saccular development.

Unlike the observations that have been reported in mice, *cloche* zebrafish mutants, in which endothelial cells are almost completely ablated (Liao et al., 1997), do not exhibit any defects in liver budding (Field et al., 2003). However, it has been shown that endothelial cells provide instructive signals that regulate the apicobasal polarisation of hepatocytes, thereby influencing the spatial organisation of the intrahepatic biliary network (Field et al., 2003). Zebrafish *heart of glass* (Mably et al., 2003) and *valentine* (Mably et al., 2006) mutants, which contain mutations in Heg and Ccm2 genes respectively, have disrupted vasculature, defective hepatocyte apicobasal polarity, and exhibit aberrant intersection of the hepatic biliary and vascular networks (Sakaguchi et al., 2008). Heg encodes a transmembrane protein that interacts via its intracellular domain with the CCM signalling complex, of which Ccm2 exists as a scaffold protein (Mably et al., 2006). Both proteins are expressed by endothelial cells, suggesting an endothelial role for this receptor complex in directing hepatocyte polarisation. A recent study in which Heg was conditionally removed from liver endothelial cells in *Lyve1-Cre; Heg* ^*flox*^ mice also demonstrated vascular and biliary patterning defects (Zhu et al., 2022). Furthermore, Heg-deficient endothelial cells had reduced expression of Wnt ligands/agonists including Wnt2, Wnt9b and Rspo3, which subsequently limited the expression of canonical Wnt-signaling target Axin2 in hepatocytes, suggesting these Wnt ligands act in an angiocrine manner to regulate liver patterning, via molecular mechanisms which remain to be further investigated (Zhu et al., 2022) (Figure 4). The role for angiocrine Wnt ligands in postnatal hepatocyte growth is further supported by reduced liver size and impaired liver zonation/patterning in *Stab2-Cre; Wls* ^*flox*^ mice, in which secretion of all Wnt ligands has been inhibited by removal of the Wnt cargo receptor (Wntless) specifically in liver endothelial cells (Leibing et al., 2018). Single cell transcriptomics has also identified the endothelial specific tyrosine kinase Tie2 receptor as a regulator of Wnt activity in vessels. (Inverso et al., 2021).

In contrast to previous work using *Flk1* ^*−/−*^ mouse embryos, some studies have suggested that endothelial cells are necessary for liver development as early as endoderm hepatic fate specification (Han et al., 2011). Compared to endoderm cultured alone, co-culture with endothelial cells significantly improved hepatic specification and expansion, which was accompanied by changes in Wnt and Notch target genes, suggesting endothelial cells may induce hepatic specification via modulation of Wnt and Notch signaling (Han et al., 2011). Activation of Wnt (Wnt3a) or Notch (NICD) signaling promoted endoderm expansion at the expense of hepatic specification, however inhibition of Wnt and Notch signaling via treatment with the Wnt inhibitor Dkk1 and Notch inhibitor GSI respectively, led to a significant enhancement of hepatic specification and reduced proliferation (Han et al., 2011). Notch signalling has also been implicated in biliary development, whereby inhibition of Notch signaling in hepatoblasts via knockout of the Notch activated transcription factor Rbpj has shown a significant reduction in the number of bile ducts (Zong et al., 2009). The Notch ligand Jag1 is highly expressed in the endothelium of the portal vein (Adams and Jafar-Nejad, 2019), and hence suggested an angiocrine mechanism of Jag1 activating Notch in adjacent hepatoblasts to promote normal biliary differentiation (Zong et al., 2009). However, further work assessing mice with conditional deletion of Jag1 in the endothelium (with VE-cadherin-Cre) or smooth muscle (with SM22-Cre) indicates that Jag1 is required in the perivascular portal vein mesenchyme, rather than endothelial cells, for bile duct morphogenesis (Hofmann et al., 2010). Taken together, this suggests that Notch signaling plays important roles in directing hepatoblast fate choice during liver development, with Notch activation promoting biliary duct differentiation, while Notch inhibition promotes hepatocyte differentiation, with the vasculature (via endothelial and/or perivascular cells) directing aspects of this process.

Other publications further highlight the essential requirement of vessels in liver development. For example, sinusoid specific deletion of GATA4 results in the conversion of traditional liver sinusoids into a continuous vascular network, leading to fibrosis, liver hypoplasia and ultimately embryonic lethality (Geraud et al., 2017). However, using several conditional knockout mouse lines, Geraud et al. showed that these defects were not the result of altered secretion of known angiocrine factors such as BMP-2, HGF, Rspo3 or Wnt ligands, and is likely caused by GATA4 control of endothelial cell junctional stability (Geraud et al., 2017).

### Pancreas

The pancreas is a critically important gastrointestinal organ that is unique in its ability to perform both exocrine and endocrine functions (Rush, 1806; Da Silva Xavier, 2018). In mice, pancreatic development is first observed when the dorsal endoderm begins to evaginate into the surrounding mesenchyme, opposite to the hepatic outgrowth, which is followed by protrusion of the ventral bud from the ventral foregut endoderm (Kim et al., 1997; Kumar et al., 2003). Importantly, the various processes involved in pancreatic embryology coincide with the development of local vasculature in a manner reminiscent of coordinated growth (Lammert et al., 2003; Cleaver and Dor, 2012).

Early budding of the pancreatic rudiment occurs at highly specific locations in the foregut endoderm which are intimately associated with the nearby endothelium of the dorsal aorta and vitelline veins (Lammert et al., 2003). Evagination of these pancreatic domains, marked by Pdx1 expression, only begins after endothelial contact, with the close pancreatic-endothelial interaction persisting throughout islet formation and maintained well beyond embryogenesis (Lammert et al., 2001).

At the earliest stages of pancreas development, vessels have been found to promote tissue formation. In *ex vivo* cultured mouse embryonic endoderm, the gut tube continues to develop while pancreatic formation is impaired; however when the same tissue is co-cultured with the dorsal aorta or other tissues containing endothelial cells, pancreatic-like structures are formed, marked by expression of the master regulator of pancreatic development Pdx1, and the endocrine cell differentiation marker, insulin (Lammert et al., 2001). Additionally, in *xenopus* embryos lacking a dorsal aorta via mechanical removal of lateral plate angioblasts, expression of insulin and markers of the dorsal pancreatic anlage including *NeuroD* and *Pax6* were significantly reduced, thereby demonstrating an important role of endothelial cells in pancreatic formation (Lammert et al., 2001). To assay later-stage postnatal pancreas development, Lammert et al. overexpressed VEGF under control of the Pdx1 promoter in mice, which resulted in a hyper-vascular pancreas and a significant increase in the number of pancreatic islets, at the expense of exocrine acinar cell differentiation (Lammert et al., 2001). Additionally, ectopic insulin-expressing cells were also identified in other areas including the stomach, suggesting vessels are inductive for pancreatic endocrine cell differentiation (Lammert et al., 2001). Other work using *Flk1* ^*−/−*^ mice which lack endothelial cells (Yamashita et al., 2000), demonstrated that while the initial induction of endodermal cells positive for Pdx1 does not require the aorta or endothelial cell interactions, emergence of the pancreatic bud and maintenance of Pdx1 expression does require endothelial cells (Yoshitomi and Zaret, 2004). Additionally, aortic endothelial cell contacts were shown to be important for dorsal pancreatic progenitor development and induction of Ptf1a, another pancreatic marker, while endothelial cells of the vitelline veins did not possess the same functions for inducing ventral pancreatic progenitors (Yoshitomi and Zaret, 2004). Adding complexity, another study showed that aortic endothelial cells promote survival of nearby Isl1-positive dorsal mesenchymal cells, that in turn signal to the dorsal pancreatic bud via FGF10 to promote maintenance of Ptf1a expression (Jacquemin et al., 2006). To date, the signals from aortic endothelial cells that drive its angiocrine function in pancreatic development remain unknown.

Cross-talk signals from the early pancreatic endoderm to embryonic endothelium are also important for pancreatic development. In chick embryos, the pancreatic endoderm was reported to secrete the chemokine CXCL12 (also known as SDF1), which attracts angioblasts expressing chemokine receptor type 4 (CXCR4) (Katsumoto and Kume, 2011). Endoderm cells in direct contact with angioblasts then initiate expression of Pdx1 (Katsumoto and Kume, 2011). VEGF-A expression in the pancreas has also been shown to direct appropriate vascularisation, which in turn affects pancreas development. One study showed loss of VEGF-A leads to impaired islet vascularisation, reduced β-cell proliferation and ultimately impaired glucose clearance (Reinert et al., 2013). However another group reported that ablation of VEGF-A leads to excessive acinar differentiation and proposed that the organisation of endothelial cells around pancreatic branches regulates typical acinar differentiation (Pierreux et al., 2010).

*In vitro* co-culture studies have attempted to address the interactions between endothelial cells and pancreatic progenitors. Embryoid bodies derived from human pluripotent stem cells were co-cultured with endothelial cells, to assess effects on differentiation of insulin-producing pancreatic β-cells, which exhibited enhanced insulin secretion *in vitro*, as well as *in vivo* upon transplantation into mice (Talavera-Adame et al., 2016). Talavera-Adame et al. suggest enhanced BMP signaling may drive the pancreatic cell differentiation, although it is not clear if BMPs are produced by the endothelial or pancreatic components of the co-culture (Talavera-Adame et al., 2016).

In contrast to their roles in endocrine cell differentiation, endothelial cells have also been implicated in pancreatic progenitor cell self-renewal. In *in vitro* studies, human embryonic stem cell-derived PDX1 +ve pancreatic progenitor cells exhibited increased proliferation and impaired differentiation towards hormone-expressing cells when co-cultured with endothelial cells (Kao et al., 2015). Through expression screening, EGFL7 was identified as a candidate secreted angiocrine factor. Knockdown of EGFL7 in endothelial cells with shRNAs inhibited the ability of the endothelial cells to promote pancreatic progenitor proliferation, and addition of recombinant EGFL7 was able to expand the pool of pancreatic progenitors (Kao et al., 2015). Overexpression of EGFL7 specifically in endothelial cells under control of the Tie2 promoter in an *in vivo* mouse model caused pancreatic buds to appear larger at E9.5-10.5, with increased proliferation, while at E15.5 there was a reduction in differentiated insulin + ve endocrine cells, consistent with the *in vitro* hESC studies (Kao et al., 2015). Taken together, this implies endothelial cells can play differential stage-dependent roles in pancreatic progenitor cell specification, progenitor maintenance, and endocrine differentiation.

N-Cadherin knockout (*Cdh2* ^*−/−*^) mice lack formation of the dorsal pancreas, owing to failure of dorsal pancreatic mesenchymal cell survival (Esni et al., 2001). *Cdh2* ^*−/−*^ embryos die during early embryogenesis due to cell adhesion defects in the heart, however restoration of N-cadherin expression in the heart driven with the cardiac-specific *a* myosin heavy chain promoter allows embryos to survive until E11.0 due to rescued heart and vascular function (Luo et al., 2001). Surprisingly, pancreas formation was restored in these cardiac-rescued embryos, indicating N-cadherin does not play a cell-autonomous role in pancreatic mesenchymal survival, but rather pancreatic agenesis is a result of attenuated cardiovascular function and development (Edsbagge et al., 2005). Using *ex vivo* cultures of foregut explants, impaired pancreas formation in *Cdh2* ^*−/−*^ tissues could be rescued with either plasma or sphingosine-1 phosphate (S1P) (Edsbagge et al., 2005). However, as S1P is primarily expressed by haematopoietic cells (Yatomi et al., 1995) these results suggest a blood-born origin of this signal rather than an angiocrine source. In exploring the sphingosine signaling pathway further with the use of S1P receptor knockout mice (*S1P1* ^*−/−*^) additional roles were uncovered for vasculature in later stages of pancreas branching and morphogenesis. S1P1 is specifically expressed in endothelium (Kono et al., 2014), and there is excessive vessel formation in *S1P1* ^*−/−*^ mice (Sand et al., 2011). Unexpectedly, the size of the dorsal pancreas is reduced in *S1P1* ^*−/−*^ mice, due to reduced proliferation of Pdx1^+^ progenitors, suggesting that blood vessels may restrict the expansion of the pancreas at later stages of development (Sand et al., 2011). Indeed, this notion was supported by the finding that ablation of endothelial cells in an explant model leads to significant expansion of the pancreatic epithelium (Sand et al., 2011). Further evidence to support this conclusion comes from forced hypervascularisation via Pdx1-specific overexpression of VEGF, which also restricted development of the pancreas by impairing branching and differentiation of endoderm towards both endocrine and acinar fates at E12.5 (Magenheim et al., 2011). Conversely, when Magenheim et al. removed vessels in an explant model, they also observed an increase in organ size (Magenheim et al., 2011). The findings from VEGF overexpression in mice conflict with the previous work of Lammert et al. which instead described an increase in endocrine differentiation. This may be due to analyses being performed at differing ages (i.e., E12.5 [Magenheim et al., 2011) and adult mice (Lammert et al., 2001)]. While there has been some speculation, future studies should aim to reconcile the essential paracrine role of endothelial cells in early pancreas development with the negative regulation of branching and epithelial growth seen at later ages.

### Lung

Blood vessels constitute an integral component of the lung alveoli, facilitating delivery of oxygen to tissues while also removing carbon dioxide via gas-exchange between alveolar epithelial cells and capillary endothelial cells through the thin alveolar membrane (Mammoto and Mammoto, 2019). Blood vessels are also intimately associated with lung alveolarization during development. Early evidence of a role for blood vessels in normal lung development was shown through treatment of newborn rats with anti-angiogenic agents such as thalidomide, fumagillin and the VEGFR2 inhibitor SU-5416, which decreased pulmonary arterial density and alveolarization (Jakkula et al., 2000). Similarly in mouse, intrauterine injection of anti-VEGFR2 neutralizing antibodies, or genetic knockout of specific isoforms of VEGF caused respiratory distress and lung maturation defects in preterm-born pups (Compernolle et al., 2002). Overexpression of VEGF in bronchial epithelial cells also caused respiratory distress in newborn pups, demonstrating precise control of VEGF levels are essential for correct lung vascularization and bronchial morphogenesis (Akeson et al., 2005). Inhibition of endothelial cell function, without reducing endothelial cell numbers in the lung via disruption of PECAM-1 also caused impaired alveolarization (DeLisser et al., 2006), suggesting it is not merely the loss of endothelial cells that inhibits alveolarization, but that endothelial cells may play functional roles in promoting lung development. Blood vessels have also been implicated in patterning of the branched epithelial airways during early lung development, a process shown to be independent of simply a perfusion role for vessels in tissue development (Lazarus et al., 2011), further suggesting angiocrine mechanisms may be responsible for directing epithelial branching.

Suggestions of cross-talk between endothelial and epithelial cells in the developing lung arose through studies in endothelial NO synthase (eNOS) knockout mice, which exhibit defective pulmonary development, in part attributed to defective vascular development, thickening of the saccular septal and defective surfactant production (Han et al., 2004). However, given eNOS is expressed by both lung endothelial and epithelial cells, it was unclear whether NO may be impacting lung epithelial cell maturation via angiocrine or autocrine roles (Han et al., 2004). Further evidence for an angiocrine role for NO came from a recent study demonstrating endothelial Sash1 is required for activation of eNOS and production of NO by the endothelium, with endothelial-specific knockout of Sash1, and hence endothelial loss of NO production, leading to deficient alveolar epithelial cell maturation (Coulombe et al., 2019). Furthermore, defective alveolar epithelial cell maturation in Sash1 knockout embryos could be rescued by treatment of pregnant dams with a nitric oxide donor (SNAP) or by activation of downstream NO signalling with the soluble guanylate cyclase activator Riociguat (Coulombe et al., 2019). Future work is needed uncover the mechanisms of how angiocrine NO signalling to alveolar epithelial cells leads to upregulation of maturation and surfactant associated transcripts.

**FIGURE 5:**
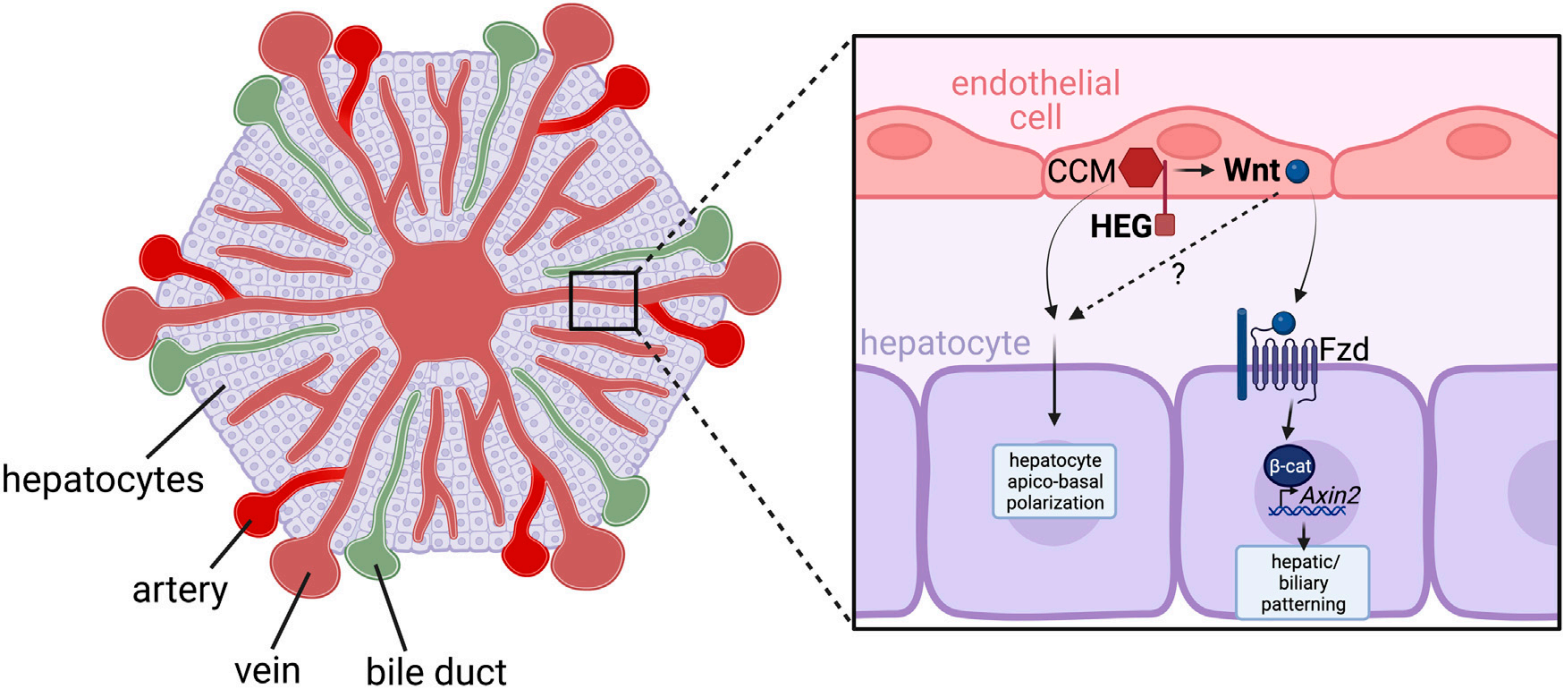
HEG/CCM-induced angiocrine Wnt signaling regulates hepatic vasculature and biliary network patterning. Heart-of-glass (HEG) is a transmembrane protein that engages with the intracellular cerebral cavernous malformation (CCM) complex, which regulates the angiocrine expression of Wnt ligands such as Wnt2 and Wnt9b in liver endothelial cells. These angiocrine Wnts signal to Frizzled (Fzd) receptors on adjacent hepatocytes, which stimulates canonical Wnt/β-catenin signaling pathway activity and expression of the target gene Axin2. While the mechanisms have not yet been fully elucidated, this activation of canonical Wnt signaling in hepatocytes is essential for correct patterning of the hepatic biliary and vascular network. Furthermore, HEG/CCM has also been shown to regulate hepatocyte apico-basal polarity, however whether this is also dependent of secretion of angiocrine Wnts remains to be determined.

In agreeance with a critical role for VEGF in lung development, conditional removal of VEGF specifically in lung epithelial cells resulted in almost complete absence of pulmonary capillary development, and associated defects in saccular septation (Yamamoto et al., 2007). Interestingly, HGF expression was lowered in the lungs of these embryos, suggesting endothelial cells may be a source of HGF (Yamamoto et al., 2007). Furthermore, conditional inactivation of the HGF receptor c-Met in lung epithelial cells resulted in impaired saccular development, much like conditional inactivation of VEGF, suggesting endothelial cells may be a source of HGF for normal septal formation (Yamamoto et al., 2007). This theory was further supported by a recent study in which knockout of the BMP antagonist Matrix Gla Protein (MGP) caused aberrant hepatic differentiation of lung epithelial cells via a complex endothelial-epithelial signaling loop (Yao et al., 2017). Loss of MGP results in increased BMP-4 activity, which induces VEGF and c-Met in epithelial cells and VEGFR1 in endothelial cells (Yao et al., 2017). This in turn leads to enhanced VEGF signalling through VEGFR1 in endothelial cells which induces excessive HGF expression, which then signals to c-Met on epithelial cells, promoting their aberrant hepatic differentiation (Yao et al., 2017). Impressively, endothelial-specific deletion of HGF in MGP knockout mice was able to limit the ectopic hepatic differentiation in the lungs (Yao et al., 2017). This suggests a critical balance of angiocrine HGF is necessary to promote correct lung epithelial differentiation, without aberrant hepatic differentiation (Figure 5).

Retinoic acid (RA) has also been implicated as an angiocrine factor in lung development. In *ex vivo* cell culture assays using pulmonary endothelial cells and lung fibroblasts, VEGF was shown to stimulate expression of retinoic acid synthesising enzymes (RALDHs) and RA in endothelial cells, and in turn RA was shown to stimulate expression of FGF-18, which regulates elastin production, in lung fibroblasts (Yun et al., 2016). Furthermore, inhibiting RA synthesis in newborn mice with disulfiram impaired alveolar development with decreased FGF-18 and elastin expression, while treating rats with impaired alveolar development (due to VEGF signaling inhibition) with RA and vitamin A partially prevented alveolarization defects (Yun et al., 2016). Another recent study identified the RA catabolizing enzyme, Cyp26b1, as highly enriched in lung endothelial cells throughout development, with complete knockout leading to defects in distal alveolar development and epithelial cell differentiation (Daniel et al., 2020). Additionally, treatment of pregnant dams with RA caused lung defects in wildtype and Cyp26b1^+/−^ embryos, to a similar extent as those observed in untreated Cyp26b1^−/−^ embryos, suggesting that limiting RA signalling in late gestation is important for distal airway development (Daniel et al., 2020). These studies further highlight the importance of striking the correct balance of angiocrine signalling to elicit the appropriate developmental outcome.

Endothelial-derived MMP14 has also been shown to drive release of matrix-associated EGFR ligands that are essential to promote regenerative alveolarization after lung injury (Ding et al., 2011), as well as to support growth of airway basal cells in culture (Ding et al., 2015), however a role for angiocrine MMP14 in embryonic lung development remains to be investigated.

### Kidney

The fully functional kidney is made up of multiple filtering modules called nephrons that filter and purify blood. Filtration within each nephron is accomplished in the glomeruli which comprises a highly stereotypical arrangement of specialised vessels sitting in close association with mesangial cells (specialised pericytes), podocytes (specialised epithelia) and a basement membrane (Khoshdel Rad et al., 2020). In addition to endothelial cells being essential for the physiological functions of the kidney, they also play critical roles in glomeruli formation. Following specification of podocytes from mesenchymal precursors these cells express VEGF and class 3 semaphoring to regulate recruitment of endothelial cells and vascular ingrowth (Reidy et al., 2009). In the absence of endothelial cells, such as in the zebrafish *cloche* mutant, podocytes are able to form a basement membrane, but their structure and maintenance are affected (Majumdar and Drummond, 1999). Blood flow within vessels has also been shown to be important for their sprouting into the glomerular primordia (Nishimura et al., 2022) and for glomerular morphogenesis (Serluca et al., 2002). Blood flow was found to regulate the expression of the stretch-responsive gene matrix-metalloproteinase-2 (MMP-2), with blockade of MMP-2 activity in zebrafish inhibiting glomerula formation (Serluca et al., 2002). This finding therefore suggests that MMP-2 secreted from endothelial cells may act to alter matrix composition or to unlock the activity of other growth regulators located outside of the cells which remain to be identified (Serluca et al., 2002). As in other organ systems endothelial cells within the kidney express PDGFB to recruit PDGFRβ expressing accessory cells. Mesangial cells fail to align the vasculature in mice lacking PDGFB from endothelial cells which leads to glomeruli malformation (Bjarnegard et al., 2004). More recently, molecular characterisation of single endothelial cells from mouse kidneys has uncovered a large degree of heterogeneity, with the identification of 7 major endothelial cell clusters that represent different vascular sub-types which dynamically change expression profiles throughout development (Barry et al., 2019). These new genome-wide datasets identify many potential angiocrine factors that could regulate kidney development that require functional validation *in vivo*.

### Nervous system

Coordinated development of the neuronal and vascular systems has been appreciated since the first anatomical description of the mammalian body plan which described precise alignment of major axonal tracts alongside the major blood vessels (Carmeliet and Tessier-Lavigne, 2005). One of the first documented roles for paracrine signalling from blood vessels was the description of the dorsal aorta promoting the differentiation of neural crest progenitor cells into autonomic neurons of the peripheral nervous system (Reissmann et al., 1996). After exiting the neural tube, a subset of neural crest cells migrate along blood vessels and within the somites toward the dorsal aorta to seed the sympathetic ganglia (Ruhrberg and Schwarz, 2010). Several members of the Bone Morphogenetic Protein (BMP) family are expressed and secreted by endothelial cells in the dorsal aorta to direct sympathetic neuron differentiation (Reissmann et al., 1996). Detailed analysis of this process refined the angiocrine role of BMPs, showing that in addition to their direct roles in specifying specific differentiation paths, BMPs also promote expression of the chemokine stromal cell-derived factor-1 (SDF -1) and Neuregulin 1 in the region around the aorta to attract the migrating neural crest cells (Saito et al., 2012).

Within the central nervous system, neural stem cells play a key role in promoting vessel growth through the expression of growth factors such as VEGF and WNT family members (Hogan et al., 2004; Reis and Liebner, 2013). In turn, vessels have been identified as contributors to the division mode (i.e., symmetric vs. asymmetric), cell cycle progression and differentiation of various cells within the central nervous system (Tata and Ruhrberg, 2018). The earliest indication that vessels influence neuronal development came from the finding that neurospheres from E10 mouse embryos expanded quicker in the presence of soluble vessel derived factors which promoted proliferation and inhibited differentiation of neural progenitors by increasing expression of Notch signalling pathway members (Shen et al., 2004). Similar roles for vessels have been described in the mouse hindbrain, where mouse models with decreased vascular density had alterations in progenitor divisions which led to an increase in neural precursor differentiation (Tata et al., 2016). These findings argue for a pro-proliferative role for vessels on neuronal precursors, however the specific angiocrine signals regulating these events have not been identified.

In other regions of the central nervous system, blood vessels have instead been proposed to positively regulate cell divisions that promote differentiation. The first study to detail this phenomenon forced excessive vessel growth in the mouse cortex by ectopic expression of VEGF which led to advanced neural precursor maturation (Javaherian and Kriegstein, 2009). Using genetic mouse models, it was also found that reduced vessel density in the cortex induced hypoxia and HIF1α expression and that this promoted neural stem cell maintenance (Lange et al., 2016). In this case the effects were related to oxygen levels and hypoxia as reduction of HIF1α normalised the differentiation of neural precursor cells (Lange et al., 2016). In addition to the proposal that blood vessels promote tissue oxygenation to regulate neurogenesis, others have proposed roles for physical associations between endothelial cells and neuronal precursors. Within the ventral telencephalon, radial glia end feet are suggested to form connections with vessels, which are important in regulating proliferation and differentiation (Tan et al., 2016). More recently it has been shown that the filopodial extensions of actively growing endothelial cells connect directly with proliferative neural precursors in the ventral telencephalon to promote their differentiation (Di Marco et al., 2020). In mice with increased filipodia the cell cycle length of neural precursors is lengthened which favours earlier differentiation (Di Marco et al., 2020). While the molecules regulating this physical association between vessels and neural precursors remain to be identified, members of the Integrin and cell adhesion molecule families are likely candidates (Proctor et al., 2005; Hirota et al., 2015; Tan et al., 2016; Hu et al., 2017; Zhang S. G. et al., 2020). Given the physical association of blood vessels with neural precursors in other brain regions (Tata and Ruhrberg, 2018), this mechanism may also play a more prominent role in regulating neurogenesis.

**FIGURE 6:**
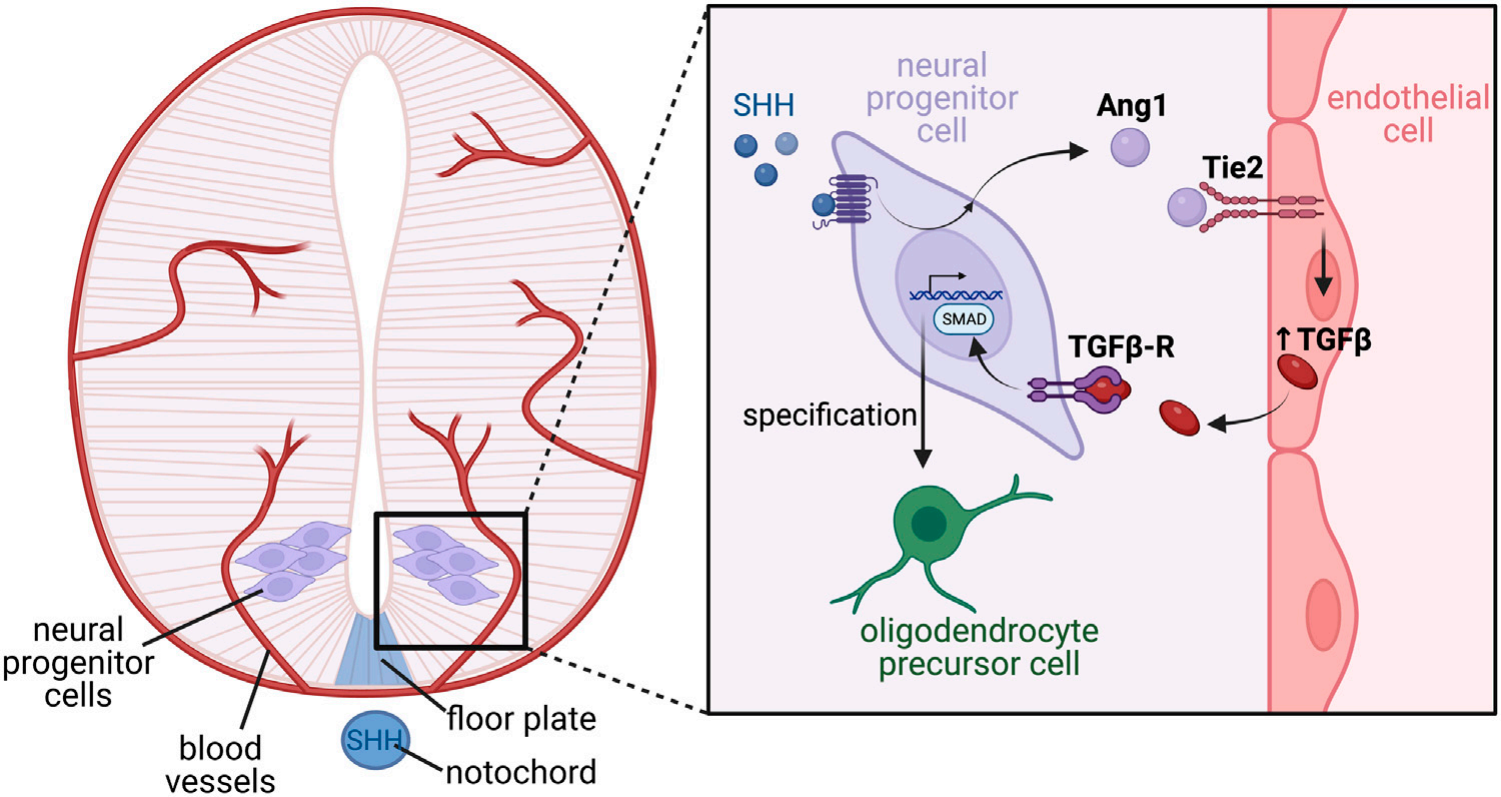
Complex tissue-endothelial cross talk mediates oligodendrocyte differentiation in the developing ventral spinal cord. The developing spinal cord encompasses multipotent neural progenitor cells, through to differentiated neurons, which are further specified into subtypes depending on their function and location along the dorsal-ventral axis. Specification is largely driven by morphogen gradients such as SHH expressed from the notochord and floor plate, which establishes patterning of the neural tube via induction of differential transcriptional programs. Blood vessels invade the developing spinal cord, and are a source of angiocrine factors which also promote correct neuronal specification. In response to SHH, neural progenitor cells located within the motor neuron progenitor domain of the ventral spinal cord express the ligand angiopoietin 1 (Ang1) which acts on Tie2 receptors located on endothelial cells. Tie2 signalling in the endothelial cells stimulates angiocrine release of TGFβ, which acts in a paracrine manner on its receptor, TGFβ-R, located on the neural progenitor cells. Active TGFβ-R signalling stimulates SMAD phosphorylation, which activates a transcriptional program ultimately driving differentiation of the neural progenitors into oligodendrocyte precursor cells.

Outside of their roles on neurons, vessels also affect the development of additional cell types within the nervous system. Soluble factors from endothelial cells were found to promote growth dynamics of astrocytes from neonatal rat brains (Estrada et al., 1990) and later shown to promote the differentiation of astrocyte precursors within the developing rat optic nerve, with Leukemia inhibitory factor (LIF) found to be an angiocrine signalling component driving astrocyte differentiation in these assays (Mi et al., 2001). Finally, within the spinal cord, endothelial-derived TGFβ has recently been described as an angiocrine factor that regulates oligodendrocyte differentiation during embryonic development (Paredes et al., 2021), through a mechanism requiring crosstalk between many cell-types (Figure 6). Paredes et al. showed that neural progenitor cells within the spinal cord express the secreted glycoprotein angiopoietin 1 (Ang1) prior to oligodendrocyte formation, which may be regulated by the morphogen sonic hedgehog (SHH) derived from the notochord and floor plate. Using conditional mouse knockouts to remove Ang1 from neural precursors, or its tyrosine kinase receptor Tie2 from blood vessels, the Ang1-Tie2 signalling axis was shown to be essential for oligodendrocyte differentiation by regulating expression of TGFβ1 in endothelial cells. Furthermore, TGFβ1 signalling pathway activity was reduced in neural progenitors from conditional Ang1 knockout mice, and spinal cord explant assays demonstrated addition of recombinant TGFβ1 was able to rescue oligodendrocyte formation in explants from conditional Ang1 and Tie2 mutant mice (Paredes et al., 2021). While we await the analysis of vessel specific knockouts, TGFβ1 represents an ideal candidate as an angiocrine factor regulating oligodendrocyte differentiation.

### Haematopoiesis

Haematopoiesis is the process by which blood cells are produced. It is first observed during embryogenesis and continues indefinitely throughout life to maintain blood cell populations (Jagannathan-Bogdan and Zon, 2013). Most research has primarily focused on the role of angiocrine signalling in adult haematopoiesis, which lies outside the scope of this review. The roles of angiocrine signalling in fetal haematopoiesis is far less understood (Gao et al., 2018). In mice, haematopoietic stem cells (HSCs) begin to emerge during the third phase haematopoiesis at E10.5 via endothelial-to-haematopoietic transition in the aorta-gonad-mesonephros (AGM) region, where they will eventually migrate to the fetal liver (Jaffredo et al., 1998; Bowie et al., 2006). HSCs undergo subsequent migration to seed the fetal spleen and later the bone marrow, creating the primary haematopoietic niches for the early postnatal period (Christensen et al., 2004). Currently, observations of angiocrine signalling during fetal haematopoiesis has largely been restricted to the liver and bone marrow.

Early reports of angiocrine signalling involvement in haematopoiesis originated from studies investigating liver organogenesis, where sinusoidal endothelial cell-specific knockout of GATA4 resulted in significant disruption to the vascular architecture during liver development (Geraud et al., 2017). In addition to widespread liver defects, this mitigated the migration of blood cell progenitors, with a significant decrease in HSC and erythromyeloid cells within the liver, in conjunction with increased numbers of both cell types in the peripheral blood (Geraud et al., 2017). Furthermore, conditional deletion of stem cell factor (SCF) from both hepatic stellate cells and endothelial cells resulted in an almost complete ablation of HSCs (Lee et al., 2021). However, when SCF was conditionally removed from only one of these cell types, the ablation of the HSC population was minimised significantly (Lee et al., 2021). It is also notable that the stellate cell-specific knockout had a more significant effect on HSCs than the endothelial-specific knockout, suggesting that the angiocrine role of SCF may be dispensable for maintaining HSCs in the liver during development (Lee et al., 2021).

The advent of single-cell RNA sequencing provided a significant breakthrough in elucidating the mechanisms involved in fetal haematopoiesis. Single-cell sequencing of fetal bone marrow cells indicates that Wnt2 expression is upregulated in arterial endothelial cells of mice at E18.5, and that Wnt2 receptors Frizzled-5 and Frizzled-9 are upregulated in HSCs (Liu et al., 2022). Genetic inhibition of Wnt ligand secretion by conditional deletion of Wntless (Wls) specifically in arterial endothelial cells resulted in defective haematopoietic colonisation and reduced HSC proliferation at E18.5. Treatment of HSPCs with Wnt2 also promoted β-catenin dependent proliferation (Liu et al., 2022). This is suggestive of a role for angiocrine Wnt2 signalling in the regulation of haematopoietic cell development. Angiocrine signalling in the context of fetal haematopoiesis remains an understudied field of research, especially in comparison to other organ systems. Future work should endeavour to identify specific angiocrine factors involved in fetal haematopoiesis and to explore a potential angiocrine contribution to the other haematopoietic phases.

### Salivary gland

Endothelial cells have also been shown to influence patterning of the salivary gland epithelium. Using *ex vivo* organ culture models, depletion of endothelial cells or inhibition of VEGFR2 signalling, disrupted epithelial patterning of the gland (Kwon et al., 2017). Addition of exogenous endothelial cells, or the endothelial-derived factors IGFBP2 or IGFBP3 were able to restore the epithelial patterning (Kwon et al., 2017), suggesting these may represent angiocrine factors important for salivary gland development, that await *in vivo* validation in conditional knockout models.

## Discussion

Since Galen’s first anatomical description of blood circulation in the second century A.D. (Aird, 2011), much has been learnt about the cell-types that comprise the circulatory system and their roles in tissue function and homeostasis. The requisite nature of blood vessels was first highlighted by their necessity to regulate tissue survival through the supply of oxygen and nutrients while at the same time acting as a conduit for the removal of toxic waste products and carbon dioxide. This necessity also extends to embryonic development where vasculature has been implicated in regulating tissue morphogenesis across an expanding number of organ systems. In contrast to their roles as passive conduits for carrying blood, here we have explored the well-established and emerging roles of endothelial cells and angiocrine signals as instructors of embryonic development.

While the contribution of vessels to tissue morphogenesis is clear, and specific angiocrine factors involved in organogenesis have been identified (Table 1), many of the angiocrine factors that regulate these processes remain unknown. Molecular identification of angiocrine factors is complicated by the high degree of heterogeneity among the endothelial cells which comprise the vasculature. Such diversity underlies specialised functions of endothelial cells across different organ systems, in different organ compartments and throughout progressive developmental stages. This phenomenon has rendered the use of large-scale omics with common resources such as cell lines [i.e., HUVEC, e.g., (Wei et al., 2019; Liu et al., 2021)] and primary tissue explants [i.e., dorsal aorta, e.g., (Kalluri et al., 2019)] intractable for angiocrine factor discovery. While new advances that increase the sensitivity of proteomics approaches offer hope of identifying vessel secreted factors from limited tissue samples, single-cell sequencing approaches are already providing fruitful information for angiocrine data mining, e.g., (Paik et al., 2020; Inverso et al., 2021). The combination of various bioinformatics packages [e.g., CellChat (Jin et al., 2021)] with single-cell datasets to identify ligand-receptor pairs (and their downstream signaling readouts) specific to endothelial cells and their target cell-types has provided a highly powerful technique to develop angiocrine hypotheses. Single-cell sequencing [e.g., refs (Jambusaria et al., 2020; Paik et al., 2020; Li et al., 2021; Wang et al., 2022)], as well as high-resolution imaging (Chen et al., 2021) approaches have also uncovered the complexity of expression profiles from different endothelial cell-types between and within single organ systems, and further highlight the likelihood that angiocrine signaling requires a combination of multiple factors expressed by endothelial cells. Additionally, perivascular cells may also be responsible for producing signalling molecules that instruct organogenesis [e.g., (Hofmann et al., 2010)], calling into question the definition of an angiocrine factor; can any factor produced by a blood vessel be considered an angiocrine factor? Or must angiocrine factors be defined as those specifically produced from the endothelium? Given the diversity of cell-types that may associate with endothelial cells, for example, smooth muscle cells, pericytes, mesenchymal cells and astrocytes, we propose that the definition of an angiocrine factor should be restricted to molecules produced by endothelial cells. However, the endothelium encompasses a wide variety of phenotypes and functions depending on the particular organ system or vascular bed, including microvascular capillaries through to major arteries and veins, lymphatics, and the specialised endothelium lining the heart. Given the emerging discoveries of the vast heterogeneity of endothelial cell-types, even within individual organs, in future further sub-classification of angiocrine factors depending on vessel type or organ system may be a desirable step forward in the field.

A bottleneck in the field is the provision of *in vivo* proof that the factor(s) under investigation are derived from the endothelium, and that their removal specifically from the endothelium regulates the biological process in target cell-types. Conditional mouse models taking advantage of endothelial-specific Cre expression, such as *Cdh5-CreERT2* and *Tie2-Cre* mouse lines, have been used in many studies to uncover and explore the roles of angiocrine factors. However, a potential caveat of this approach, which is not always controlled for, are the cell-autonomous changes to gene expression and function resulting from gene deletion, Cre expression and treatment with activators such as Tamoxifen (Brash et al., 2020). Expanding the toolset of Cre drivers to remove genes from only specific endothelial cell sub-types, rather than all endothelium, will be important to move the field forward. Another resource that will likely aid this discovery process is the advancement of cell-specific gene manipulation in other model species such as zebrafish, which have rapidly progressed since the introduction of TALEN and CRISPR mediated genetic modifications.

While this review has focused on the discussion of angiocrine factors in embryonic development, it should also be noted that angiocrine and lymphangiocrine signaling has important roles in regeneration, homeostasis and disease, which have been explored in other recent reviews (Rafii et al., 2016; Pasquier et al., 2020; Gomez-Salinero et al., 2021; Chen and Ding, 2022; Liu and Li, 2023). As the molecular mechanisms regulating embryonic development are often reinitiated in regeneration, and hijacked by disease, the knowledge garnered on angiocrine factors in embryonic development is likely to impact our understanding of angiocrine signals and their potential therapeutic applications in these fields.
